# Types and Performances of Polymer Gels for Oil-Gas Drilling and Production: A Review

**DOI:** 10.3390/gels8060386

**Published:** 2022-06-17

**Authors:** Shaofei Lei, Jinsheng Sun, Kaihe Lv, Qitao Zhang, Jingbin Yang

**Affiliations:** 1 School of Petroleum Engineering, China University of Petroleum (East China), Qingdao 266580, China; 13061429096@163.com (S.L.); lvkaihe@upc.edu.cn (K.L.); atom19980818@163.com (Q.Z.);yangjingbin2018@163.com (J.Y.); 2 CNPC Engineering Technology R&D Company Limited, Beijing 102206, China

**Keywords:** polymer gel, high temperature resistance, high salt resistance, mechanical property, oil-gas drilling and production

## Abstract

Polymer gels with suitable viscoelasticity and deformability have been widely used for formation plugging and lost circulation control, profile control, and water shutoff. This article systematically reviews the research progress on the preparation principle, temperature resistance, salt resistance, and mechanical properties of the ground and in situ crosslinked polymer gels for oil-gas drilling and production engineering. Then, it comparatively analyzes the applicable conditions of the two types of polymer gel. To expand the application range of polymer gels in response to the harsh formation environments (e.g., high temperature and high salinity), we reviewed strategies for increasing the high temperature resistance, high salt resistance, and rheological/mechanical strengths of polymer gels. This article provides theoretical and technical references for developing and optimizing polymer gels suitable for oil-gas drilling and production.

## 1. Introduction

Polymer gels are elastomers with a three-dimensional (3D) network structure composed of polymers and crosslinkers as the primary agents and other additives. The 3D network structure of polymer gel can binds a large number of solvents and make it resistant to high formation pressure. As a result, polymer gels are polymer materials that have adjustable strength and suitable deformability and pumpability [1,2,3], which makes them widely used in oil-gas engineering, such as formation plugging, lost circulation control, profile control, water shutoff, fracturing during production, and wellbore strengthening during oil-gas drilling.

Polymer gels can achieve a greater operating depth than conventional packers during profile control and water shutoff operations [4,5,6] and can overcome the size-matching difficulties of conventional rigid materials in lost circulation control operations during drilling. Crosslinked polymer acids have the advantages of both conventional acids and guar gum fracturing fluids in fracturing operations, resulting in smaller friction and higher sand-carrying capacities [7,8]. Therefore, polymer gels have been very effective in various field applications of the oil-gas drilling process [9,10,11].

The polymer gels in oil-gas drilling and production engineering can be categorized into ground crosslinked polymer gel systems and in situ crosslinked polymer gel systems. The gelation process of ground crosslinked polymer gels occurs before injection, while the crosslinking of in situ crosslinked polymer gels occurs in the formation. The main advantage of ground crosslinked polymer gels is their ability to migrate at depth, while in situ crosslinked polymer gels have advantages of suitable mechanical strength and temperature resistance. Because both have advantages and disadvantages, the appropriate crosslinking system should be selected according to the specific field conditions [12,13].

Polyacrylamide (PAM) is the most commonly used polymer for preparing gels, including in situ and ground crosslinked polymer gels. PAM is inexpensive, has numerous carboxyl and amide groups, and can be hydrolyzed under high-temperature or alkaline conditions to form stable partially hydrolyzed PAM (HPAM). The presence of carboxyl and amide groups ensures that they can crosslink well with various organic/inorganic crosslinkers, forming a gel system with suitable mechanical properties [14]. The most common inorganic crosslinkers are high-valent ion crosslinkers such as Cr^3+^ and Al^3+^. Metal ion crosslinkers are inexpensive and have achieved suitable results in field applications. However, gel systems that use metal ions as crosslinkers are often only suitable for reservoirs below 80 °C, and ionic crosslinkers have nonnegligible toxicity; thus, their applications in high-temperature reservoirs are limited. Commonly used organic crosslinkers include phenolic and polyethyleneimine (PEI), which have better temperature resistance and mechanical properties than inorganic crosslinkers due to their stronger covalent bonds, and as a result, have a wide range of applications and prospects [15,16].

Polymer gels in oil-gas reservoirs are often subjected to high temperatures and salinity, and excessive temperatures and salinity can destroy the structural integrity of the polymer chains, resulting in substantial instability. Therefore, maintaining the suitable mechanical properties of polymer gels under high-temperature and high-salinity conditions is extremely difficult [17,18,19]. Researchers have optimized polymer gel properties from three aspects: polymer structure, crosslinker type, and the addition of other additives. Conventional PAM is prone to excessive hydrolysis at high temperatures, affecting the gels’ stability in the formation. Modified PAM (e.g., hydrophobically modified and amphoteric PAM) or multi-copolymers (e.g., 2-acrylamide-2-methylpropanesulfonic acid (AMPS) copolymerized with acrylamide (AM)) can optimize the temperature resistance, salt resistance, and mechanical properties of the gels. The addition of nanoparticles into the polymer gels can also create larger specific surface areas, resulting in better gel performance in high-temperature and high-salinity environments [20,21,22].

This paper systematically reviews research progress on the preparation principle, temperature resistance, salt resistance, and mechanical properties of the ground and in situ crosslinked polymer gels for oil-gas drilling and production engineering. We then comparatively analyze the applicable conditions of the two types of polymer gels. Second, we elaborate upon strategies for increasing the high-temperature resistance, high salt resistance, and gelation mechanical strength properties of polymer gels to improve the polymer structures and types, crosslinkers, and additives.

## 2. Types of Polymer Gels Used for Oil-Gas Drilling and Production

Polymer gel materials are highly flexible, viscoelastic materials that can adapt their shape to enter fractures and pore channels of different sizes and morphologies. Therefore, they have been widely used in profile control, water shutoff, lost circulation control, and fracturing in formations. Commonly used polymer gels can be categorized according to their gelation method and mainly include in situ crosslinked gelation and ground pre-crosslinked gelation. In situ crosslinked gel is sensitive to pH, temperature, and salinity variations and reduces the permeability of fractures or channels. Pre-crosslinked gelation can only be used for those reservoirs with extreme high-permeability contrast.

### 2.1. In Situ Polymer Gels Crosslinked

The gel of an in situ crosslinked gelation polymer refers to the injection of a polymer (or monomer), crosslinker, and initiator into the target formation, forming an aqueous solution that undergoes crosslinking in the formation environment and creating a viscoelastic gel with a 3D network. In situ crosslinked gels often have excellent gel strength and are adapted to plugging pores and fractures in the formation. The gel types of in situ crosslinked gel systems can be divided into rigid and weak gels, and each gel type has its applicable conditions due to its unique properties. Rigid gels have suitable strength and temperature resistance and are suitable for profile control, water shutoff, and lost circulation control near the wellbore. Weak gels of in situ crosslinked gel systems have suitable deformation and migration properties and are suitable for profile control, water shutoff, oil displacement, and fracturing in remote-well zones. Both types have achieved suitable results in field applications [23,24,25].

Acrylamide-based polymers or copolymers are the most commonly used polymers for in situ crosslinked gels and have molecular chains rich in amide and hydrolyzed carboxyl groups. Thus, the carboxyl groups can be crosslinked with metal ions (e.g., Cr^3+^ and Al^3+^) to form ionic-bonded gels. The first in situ crosslinked gel system for profile control was prepared by Philips (now ConocoPhillips) in the 1970s using partial HPAM with aluminum citrate [26,27]. Sharma et al. compounded halfway HPAM polymers with a chromium acetate derivative to prepare a chromium ion-crosslinked gel water shutoff system suitable for medium-temperature reservoirs [2]. However, it had a short crosslinking time at high temperatures; thus, it could not be used for in-depth water shutoff operations in formations. In addition, the metal ion-crosslinked gels were prone to syneresis under high-temperature conditions; therefore, this type of gel was only suitable for low and medium-temperature formations (<80 °C).

**Figure 1:**
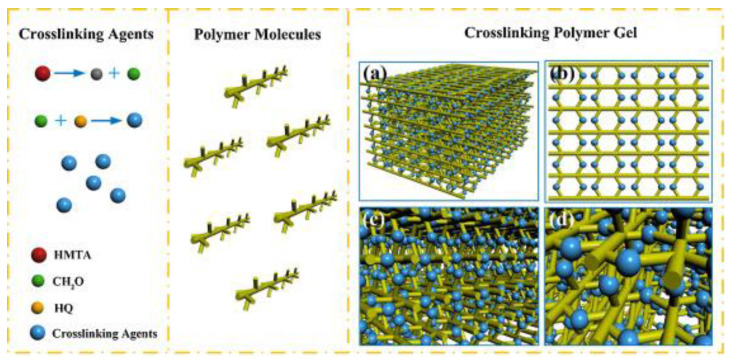
Schematic diagram showing the crosslinking mechanism of the resorcinol/HMTA gel. (**a**–**d**) Three-dimensional network structure of gels from different perspectives. (Reprinted/adapted with permission from Ref. [28]. 2016, Liu, et al.).

In addition to crosslinking with metal ions, AM-based polymers can undergo crosslinking reactions with phenolic crosslinkers due to the amide groups on the molecular chain. Commonly used phenolic crosslinkers include phenol/formaldehyde, resorcinol/hexamethylenetetramine (HMTA), and water-soluble phenolic resins. Because phenol/formaldehyde crosslinked systems are highly toxic, less toxic organic hydroquinone (HQ)/HMTA crosslinked systems are more widely used. Liu et al. studied the crosslinking mechanism as well as temperature and salt resistance of PAM/HQ/HMTA gels (Figure 1), and the results showed that the gel system had suitable salt resistance and maintained suitable stability below 140 °C [28]. In addition, covalently bonded gels produced by the reaction of organic crosslinkers with AM-based polymers will have better properties than metal ion crosslinkers and, subsequently, a wider pH and temperature adaptation range [29]. Zhao et al. prepared a gel using phenolic resin crosslinkers compounded with non-ionic PAM as an in-depth profile control agent in formations, and the system was stable at a high temperature of 143 °C and achieved suitable application results in the Xinjiang oilfield, China [30]. The weak gel oil-displacement agent, which was prepared by compounding the PAM/water-soluble phenolic resin, was applied to the Pu-125 block of the Daqing oilfield, China. The cumulative production of the whole block increased by 1400 t, comprehensive water cut decreased by 7.16%, and crude oil recovery increased by 3.58%, proving the effectiveness of the system [13].

**Figure 2:**
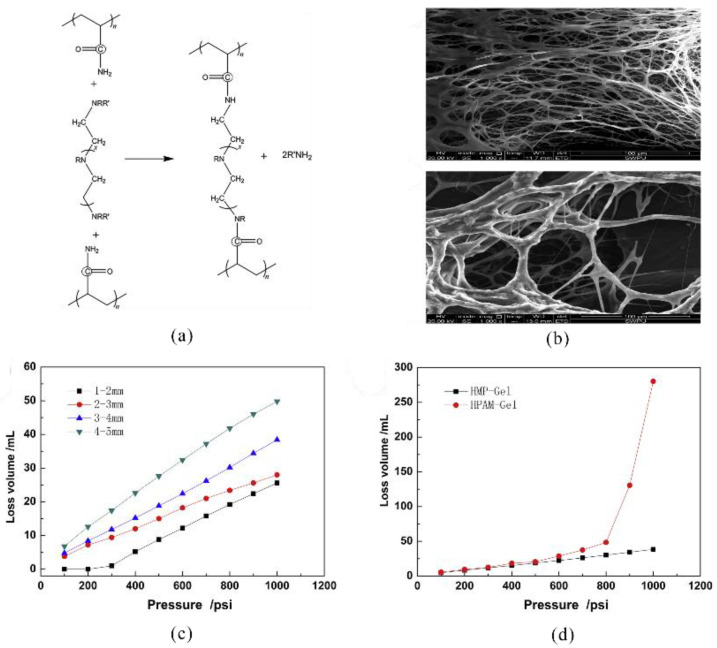
Mechanism and effect of the HMP-Gel as a lost circulation control agent. (**a**) The gelation mechanism of HMP-Gel; (**b**)the microscopic morphology of the HMP-Gel; (**c**) the performance of the HMP-Gel for different size chips bed; (**d**) comparison of plugging performance of different gel. (Reprinted/adapted with permission from Ref. [32]. 2021, Xie, et al.).

Conventional PAM is prone to degradation via chain scission at high temperatures, considerably reducing gel stability, which can be optimized through modification. PEI is an efficient high-temperature crosslinker because it can undergo the transamidation reaction with AM-based polymers, forming high-strength covalent bonds [31]. Xie et al. prepared a hydrophobically modified PAM polymer, HMP, by micellar copolymerization using AM, octadecyl acrylate (OA), and AMPS as the raw materials, where OA increased the rigidity of the polymer molecular chains and AMPS enhanced the temperature resistance of the molecular chains, crosslinking it with PEI to prepare a temperature-resistant gel as a lost circulation control agent HMP-Gel (Figure 2) [32]. This gel exhibited a controllable gelation time in the temperature range of 100–150 °C and showed an excellent plugging effect on carbonate formations [32]. In addition to the AM-based polymers, polysaccharide polymers (e.g., starch, guar gum, and xanthan gum) and their modified derivatives have been used to prepare many types of gels, and these gels are environmentally friendly but have lower temperature resistance and gel strength than AM-based gels [5].

**Figure 3:**
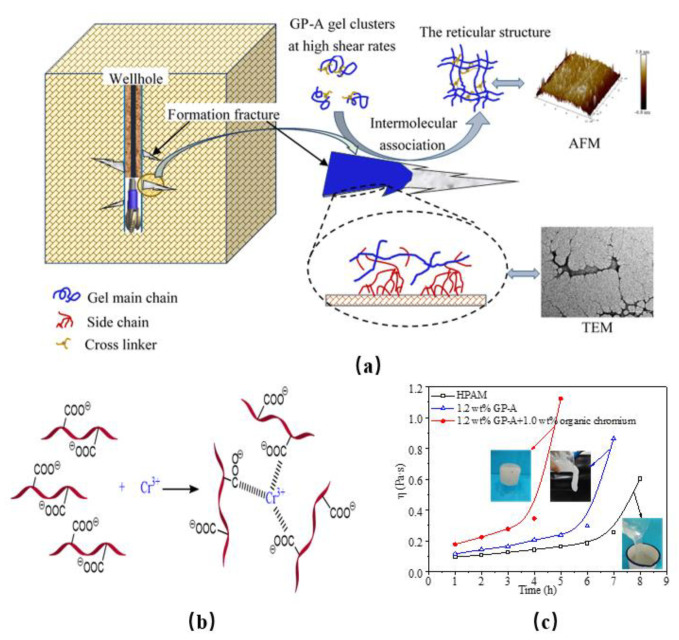
Preparation and mechanism of physically crosslinked GP-A gel as a lost circulation control agent (**a**) Schematic diagram of gel plugging in fracture; (**b**) schematic of the interaction between the GP-A gel and crosslinking agent; (**c**) gelation time of the GP-A gel under the organic chromium crosslinker [33]. (Reprinted/adapted with permission from Ref. [33]. 2021, Wang, et al.).

In addition to those mentioned above, in situ chemically crosslinked polymer gels, which require crosslinkers, and in situ physically crosslinked polymer gels have emerged in recent years that do not require the use of crosslinkers. The latter gel type is formed through the entanglement or association of polymer chains with special functional groups. Physically crosslinked gels differ from chemically crosslinked polymer gels mainly because they have unique shear thixotropy and use different crosslinking methods, giving them higher flushing resistance and better retention in fractures, making them more easily plug large fractures. To address the vicious lost circulation of drilling fluid during drilling, Wang et al. [33] developed a physically crosslinked gel plugging agent (GP-A), which mainly consisted of a spider web-type star-shaped structure, unlike the linear gel structure generated by HPAM. The GP-A gel exhibited high mechanical strength and had a strong adhesive surface; thus, it could attach to the fracture wall to increase its retention capacity (Figure 3). Based on the principle of supramolecular chemistry, Nie et al. [34] introduced hydrophobic functional groups on PAM macromolecular chains to synthesize a highly hydrophobic-associated polymer, which could spontaneously assemble through intermolecular interactions, forming a supramolecular gel with a dynamic physically crosslinked network. This gel has been used successfully for lost circulation control in more than 30 wells with a severe loss in China. Based on a similar principle, Jiang et al. [35] synthesized a gel agent for lost circulation control using a supramolecular polymer thickener (SMPT) and viscoelastic surfactant (VES) worm-like micelles. This gel had very efficient physical crosslinkers and high viscosity, strong shear thinning, and self-healing abilities at low shear rates.

In situ crosslinked polymer gels have the following advantages: (a) in situ crosslinked polymer gels used for formation plugging can generate a high-strength bulk gel in in situ pores and fractures to achieve high-strength plugging; (b) in situ crosslinked polymer gels used for oil-gas profile control and displacement have a continuous phase with suitable accessibility to formation pores and fractures of different sizes; thus, they can displace oil and gas in pores and fractures in a wide range with high sweep and displacement efficiencies; (c) in situ crosslinked polymer gels have a simple preparation and injection process but still have the following problems: (i) during the injection process, the polymer gel solution will be affected by the shearing effect of the pores in the formation, and the macromolecular chains break easily, leading to a significant reduction in the solution viscosity, which affects gelation; (ii) due to multiple components, the gel solution will exhibit chromatographic separation between the components and formation water dilution and viscosity reduction during the injection and formation migration processes, resulting in a non-uniform distribution of component concentrations, which will affect the gelation effect. In addition, (iii) compared with gels after gelation, the gel solutions before gelation are more susceptible to the influence of various factors such as high temperature and high salinity, resulting in a reduction in gel strength, which makes it challenging to achieve high-strength plugging of the formation pores and fractures. Therefore, in situ crosslinked polymer gels must be studied in depth for increasingly complex and harsh formation environments to assess their resistance to temperature, salinity, shear, and dilution.

### 2.2. Pre-Crosslinked Polymer Gels

Ground pre-crosslinked gels were developed to address the problems associated with in situ crosslinked polymer gels. The pre-crosslinked gels are formed by mixing the polymer and crosslinker in the ground equipment. Then, after crosslinking is complete, it is injected in situ, often in the form of particles, to create a plugging zone through the gel particles’ expansion, accumulation, and filling effects. Ground pre-crosslinked gels have certain advantages. Namely, they are insensitive to pH, temperature, shear, and salinity, giving them a more comprehensive range of applicable conditions. Commonly used ground pre-crosslinked gels mainly include pre-crosslinked particle gels (PPGs), polymer microspheres, and colloidal dispersion gels [19,36,37].

**Figure 4:**
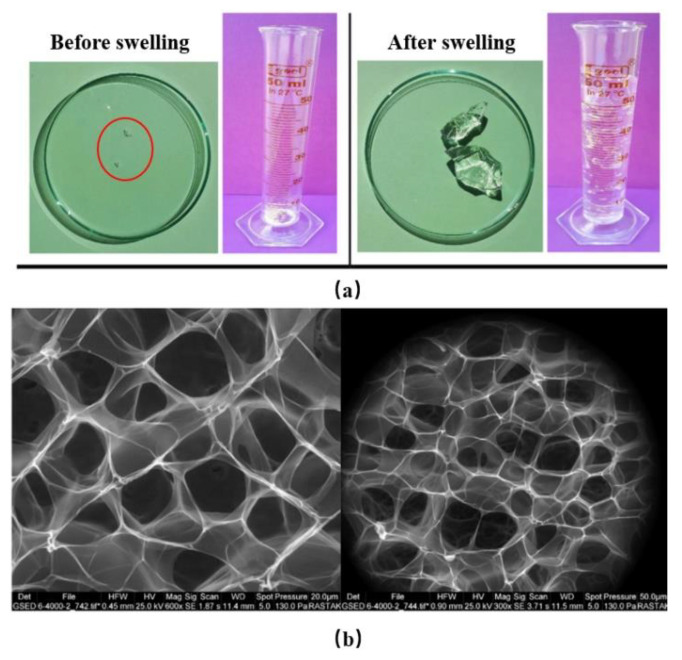
Physical photograph and microstructure of super-swelling PPG before and after swelling. (**a**) Physical photograph of super-swelling PPG before and after swelling; (**b**) microstructure of super-swelling PPG before and after swelling at room temperature. (Reprinted/adapted with permission from Ref. [40]. 2018, Lenji, et al.).

The concept of PPGs was first put forward by PetroChina in 1996. PPGs are usually made by producing a bulk gel with AM, crosslinkers, initiators, and other additives, followed by drying and crushing into particles ranging from microns to centimeters. PPG particles often contain hydrophilic groups such as amide and carboxyl groups. After dispersion in the aqueous phase, the particles absorb water and swell up to 200 times, thus achieving the high-strength plugging of the fractures. Bai et al. [38] first prepared bulk gels with AM monomers, N,N-methylenebisacrylamide (MBAA), sodium peroxodisulfate, and bentonite as the raw materials. Then, they dried, crushed, and sieved the bulk gel to obtain PPG particles, which remained stable at 120 °C for more than a year and exhibited suitable salt resistance, with practical applications for profile control in water injection wells with a salinity of 150,000 mg/L and a temperature of 107 °C. Different polymers, crosslinkers, and additives led to PPGs with different properties. In general, particle gels with a higher swelling capacity have a lower mechanical strength, while conversely, particle gels with a lower swelling capacity have a higher mechanical strength. The introduction of hydrophilic groups can enhance the swelling capacity of the PPGs. Bai et al. used AM, acrylic acid (AA), and MBAA as the raw materials to synthesize a PPG profile control agent via the free-radical method, and their study showed that the addition of AA improved the swelling capacity of the particle gel [39]. Some particular types of crosslinkers could also increase the water-absorbing capacity of the PPG. Lenji et al. prepared a super-swelling PPG water shutoff agent using sulfonated PAM powder and an aluminum nitrate nanohydrate crosslinker (Figure 4) [40]. Its swelling ratio was temperature and pH-responsive, and it could absorb water at a rate equivalent to 1000–2000 times its weight within a pH range of 5–9 while maintaining an intact 3D network structure.

**Figure 5:**
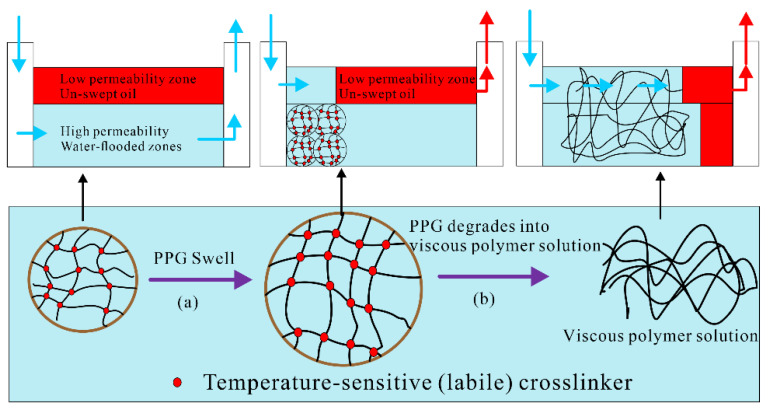
Mechanism of high-temperature stable/unstable crosslinker composite PPGs. (**a**) PPG Swells. (**b**) PPG degrades into viscous polymer solution. (Adapted from Ref. [41]. 2013, Bai, et al.).

Researchers have also studied various PPG materials with special functionality to meet the plugging needs of different formations. Bai et al. prepared a composite pre-crosslinked gel material using both high-temperature stable and high-temperature unstable crosslinkers [41]. Unlike conventional pre-crosslinked gels, the composite PPG swelled and plugged the high-permeability zone when injected into the reservoir. After a while, the unstable crosslinks of the particle gel were destroyed under the influence of reservoir temperature, and the PPG was degraded into a viscous polymer, which migrated deeper into the reservoir with the subsequent water flow, an action similar to polymer flooding; thus, it further improved the sweep efficiency and displacement efficiency (Figure 5). Pu et al. prepared a re-crosslinkable PPG (RPPG) based on an AA-AM copolymer [42,43,44]. The RPPG could swell 38 times its initial volume and re-crosslink into a high-strength bulk gel while swelling, which enhanced the ability of the particle gel layer to plug pores and fractures in the formation. Mohamadian et al. prepared a novel elastic cement designed with sufficient strength and integrity using an additive containing carbon nanotubes (CNT) in a methyl methacrylate polymer matrix. The new additive provides the appropriate rheology, dramatically reduces fluid loss, and improves cement’s mechanical properties and bonding strengths [45].

**Figure 6:**
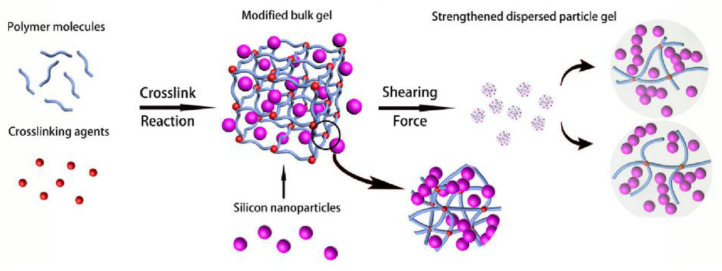
Schematic of the mechanism of stabilizing gel particles by silica nanoparticles. (Reprinted/adapted with permission from Ref. [46]. 2016, Dai, et al.).

Unlike PPG preparation, which involves first preparing the bulk gel and then mechanical dispersion granulation, Dai et al. proposed a method for preparing the dispersed particle gel (DPG) by direct mechanical shearing, where the particle gel was prepared by high-speed shearing using the polymer and crosslinker, and the particle size was controlled by the shear rate [37]. The DPG prepared using this method had a facile preparation process, had a wide adjustable particle size range, and was suitable for injection during preparation in the oilfield, which was beneficial for its promotion and application [43,44]. To optimize the performance of the DPG under high-salinity conditions, Dai et al. prepared a strengthened reinforced DPG (SDPG) with nano-silica as the reinforcing agent, resulting in better mechanical properties and viscoelasticity than conventional DPG (Figure 6) [46].

The particles of PPGs generally range from a few hundred microns to a few millimeters and can only enter formations that have large-sized high-permeability pore channels or fractures, where PPGs aim to form a high-strength plugging zone. However, due to their large size, PPG particles have a limited migration distance in formations and fail to meet the requirements of in-depth profile control or oil displacement in oil-gas reservoirs. To overcome this problem, some polymer microspheres with smaller sizes, a smooth surface, regular morphology, and suitable dispersion and suspension have been prepared. These microspheres can migrate and adsorb in the large pore channels with the dominant flow, achieve temporary plugging through the multi-particle bridge, and displace crude oil in small pores where the oil flow accumulates, thus realizing in-depth profile control and oil displacement in formations [47,48,49]. Inverse suspension polymerization and inverse emulsion polymerization are the two standard methods for polymer microsphere preparation. Yao et al. prepared micron-sized PAM elastic microspheres (MPEMs) as profile control agents via inverse suspension polymerization [47]. The microspheres showed stable performance at 100 °C, a salinity of 30,000 mg/L, and different pH levels. The field application results also showed that the microspheres could effectively improve water flooding sweep and displacement efficiencies.

**Figure 7:**
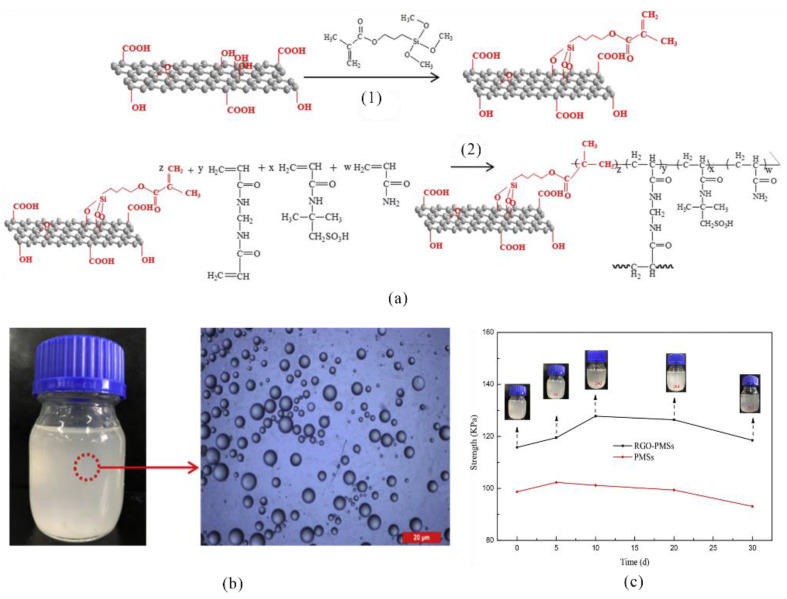
Preparation mechanism, microscopic image, and strength variations of modified GO polymer microspheres; (**1**) synthetic routes of FRGO; (**2**) synthetic routes of RGO-PMSs; (**a**) synthetic routes of GO polymer microspheres; (**b**) morphology of modified GO polymer microspheres; (**c**) strength of polymeric microspheres versus aging time [50]. (Reprinted/adapted with permission from Ref. [50]. 2020, Du, et al.).

The properties of microspheres can be optimized by introducing different materials into the polymer molecular chain. Graphene oxide (GO) has high strength and a large specific surface area. Therefore, by introducing modified GO into the structural chains of microspheres through inverse emulsion polymerization, the microspheres exhibited a significantly improved strength value of 115.8 kPa even after 30 days of aging (Figure 7), suitable retention in small pore channels, and maximum additional flow resistance of 7.12 kPa. The microspheres exhibited suitable performance for in-depth profile control and displacement in reservoirs [50]. Nanoparticles can also contribute considerably to the optimization of microsphere performance. Liu et al. used nano-silica and polymers to prepare the emulsion polymerization of organic-inorganic polymer microspheres as a profile control agent, which achieved a plugging efficiency of up to 93.87% for high-permeability zones [51].

Ground crosslinked polymer gels have the following advantages compared with in situ polymer gels. (a) Depending on the preparation process, the particle sizes of PPGs are adjustable from nanometers to millimeters or even centimeters. So, the gels can be prepared in different particle sizes according to the specific plugging requirements of different pores and fracture sizes in the formation. (b) PPGs do not undergo chromatographic separation between the different components during the injection process and are minimally affected by factors such as temperature and salinity. In addition, (c) PPGs can migrate long distances in the formation and are minimally influenced by the shearing effect of the formation pores; thus, they are suitable for in-depth plugging and oil displacement in formations. However, there are the following problems. For example, (a) the particle sizes of the PPGs must match well with the pores and fractures size in the formation; thus, PPGs have poor injectability performance for formation with pores and fractures of different sizes. (b) The PPG concentration will decrease substantially during migration under the influence of factors such as dilution of formation water, making it difficult to achieve effective blocking of in-depth pores and fractures in the formation. In addition, (c) for water shutoff or lost circulation control in formations, PPG has difficulty forming bridges between macroscopically large fractures, as well as fractures and cavities, and forming a plugging zone; thus, PPG is mainly applicable to plugging low-permeability formations with microscopic pores and fractures. Therefore, ground PPGs should be further studied to address the above problems.

## 3. Improving Temperature Resistance of Polymer Gels

With the continuous depletion of medium and shallow oil-gas resources, deep and ultra-deep oil-gas resources are gaining increasing attention. As a result, high temperatures are one of the most critical challenges faced during deep and ultra-deep oil-gas drilling and production [52,53]. Because polymers are susceptible to chain scission degradation at high temperatures, the gel strength of the polymer gel material will decrease, which affects its efficiency in these applications. This is mainly due to the dissolved oxygen in the polymer gel or the residual initiators at high temperatures, which considerably influence gel performance. Due to the presence of oxygen/residual initiators, the main chain of the gel will be prone to chain oxidation reactions at high temperatures, likely resulting in scission at weak bond points where the peroxides are generated, resulting in gel instability. In addition, the methylene groups of the crosslinked polymer gel will be subjected to nucleophilic attack at high temperatures by the protonated solvent (water), breaking and forming a hydroxyl group on one side and an amide on the other side. When the protonated solvent attacks the amide carbonyl carbon, the amide will break and generate a primary amine and carboxylic acid. Nucleophilic attack on the carbonyl carbon of the side chain amide by the protonated solvent (water) will generate carboxylic acid and ammonia. Both cleavage and hydrolysis of the above groups will destroy the 3D network structure of the gel. Numerous studies have been carried out to address the challenging problem of the high-temperature resistance of polymer gels. Currently, the main research avenues include optimizing the polymer macromolecular chain structure, optimizing the crosslinker type, and adding high-temperature-resistant additives to the gel components [54].

Commonly used polymers in oilfields, such as PAM, starch, guar gum, and xanthan gum, are prone to chain scission and are challenging to degrade at high temperatures. Introducing cyclic structures (e.g., benzene ring) or temperature-resistant groups (e.g., sulfonic acid groups) into their molecular chains by grafting and copolymerization can significantly improve the temperature resistance and tolerance of the polymer molecular chains, and, subsequently, the high-temperature stability of the gels [23]. Long et al. prepared a high-temperature-resistant pre-crosslinked gel system by polymerizing and crosslinking AM, N-vinylpyrrolidone (NVP) with a cyclic structure, and divinylbenzene (DVB); the gel strength remained stable after aging for 90 days at 130 °C [55]. AMPS is a commonly used temperature-resistant monomer for the preparation of monomer gels and/or polymers due to its long-branched chains and sulfonic acid groups. AM/AMPS copolymers have suitable thermal stability due to the steric hindrance of many methyl propane and sulfonic acid groups on the polymer chain. Chen et al. crosslinked an AM/AMPS copolymer with a phenolic crosslinker and used ethylenediamine as a crosslinking retarder to prepare a gel with a dense 3D network structure [56]. The gel maintained suitable strength and stability after 100 days of aging at a high temperature of 130 °C and was successfully used for reservoir water shutoff operations in oilfields in Northwest China. Zhu et al. prepared an in situ crosslinked gel system with suitable thermal stability and an adjustable gel strength by reacting AM/AA/AMPS terpolymers with OC-3, a novel crosslinker with hydroxyl and phenolic rings [57]. The amide groups (-CONH_2_) on the terpolymer molecular chain were crosslinked with the hydroxyl groups (-CH_2_OH) on the phenolic ring of OC-3, forming a dense 3D network structure. Scanning electron microscope (SEM) images revealed that the microscopic network gel network was approximately 10 μm in size and had a large grid thickness; thus, the system showed only minor syneresis after it was heated at 150 °C for five months. So far, the optimization of the polymer molecular structure has been studied systematically and in-depth; however, because polymer gels are composed of multiple components, studies on enhancing the high-temperature stability of gels by simply improving the temperature resistance of polymer molecules have hit a ceiling limit.

Crosslinkers are necessary components of crosslinked polymer gels. Commonly used crosslinkers for polymer gels include organic phenolics and metal ions. Metal ion-crosslinked gels are prone to hydrolysis at high temperatures, resulting in gel syneresis; however, stable covalent bonds can be formed between the amide group of the polymer and the organic crosslinker at high temperatures. Thus, gels prepared by organic crosslinkers generally have suitable temperature resistance. For monomer-crosslinked polymer gels, N,N’-methylenebisacrylamide and N-methylolacrylamide are the most commonly used crosslinkers. Chang et al. prepared a PAM gel based on resorcinol crosslinked with formaldehyde. The gel had a temperature resistance of up to 120 °C, but it was too sensitive to salt and pH. Zhuang et al. sulfonated the resorcinol used in the above gel to improve the salt and pH resistance of the gel. Unlike toxic phenolic crosslinkers, organic polymer crosslinkers such as PEI are non-toxic and environmentally friendly. The crosslinking reaction between PEI and PAM involves nucleophilic imine nitrogen on PEI replacing the amide group on PAM, forming a stable covalent bond. Therefore, PEI-crosslinked gels have better high-temperature stability than metal ion-crosslinked gels [31].

**Figure 8:**
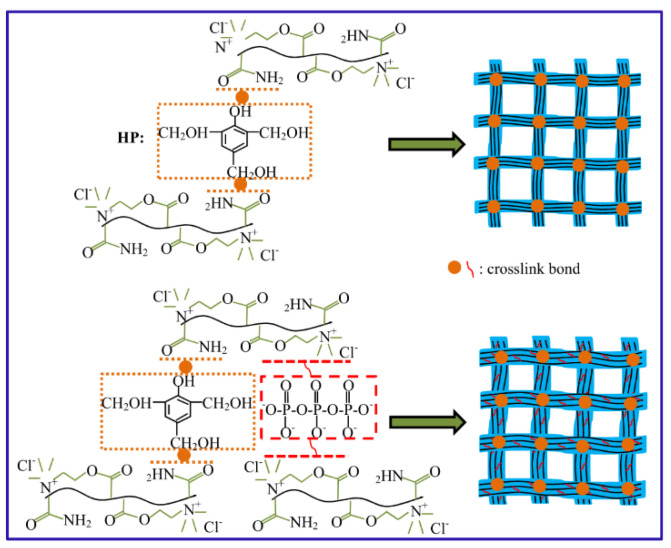
Crosslinking principles of the polymer gel with and without the addition of STPP crosslinker, respectively (Reprinted/adapted with permission from Ref. [59]. 2015, Chen, et al.).

To solve the issue of metal ion-crosslinked gels being prone to hydrolysis at high temperatures, resulting in gel syneresis, organic-inorganic composite crosslinkers are commonly used to enhance the compactness of the 3D network structure, thereby improving the high-temperature stability of the gel. Zhang et al. prepared a composite-crosslinked PAM gel system using both Cr^3+^ and phenolic resin as the crosslinkers [58]. Because the system had both weak bonds generated by the metal crosslinkers and strong bonds generated by the organic crosslinkers, the presence of the double-bond network effectively inhibited the high-temperature syneresis of the metal-crosslinked structure, which significantly improved the temperature resistance of the gel, allowing it to remain stable when aged at 140 °C for 120 days (Figure 8). Similarly, an organic-inorganic composite crosslinker composed of a phenolic resin and sodium tripolyphosphate (STPP) was crosslinked with an acryloyloxyethyl trimethyl ammonium chloride/AM (DAC/AM) copolymer to prepare a double-group crosslinked hydrogel that was capable of operating at 130 °C [59].

**Figure 9:**
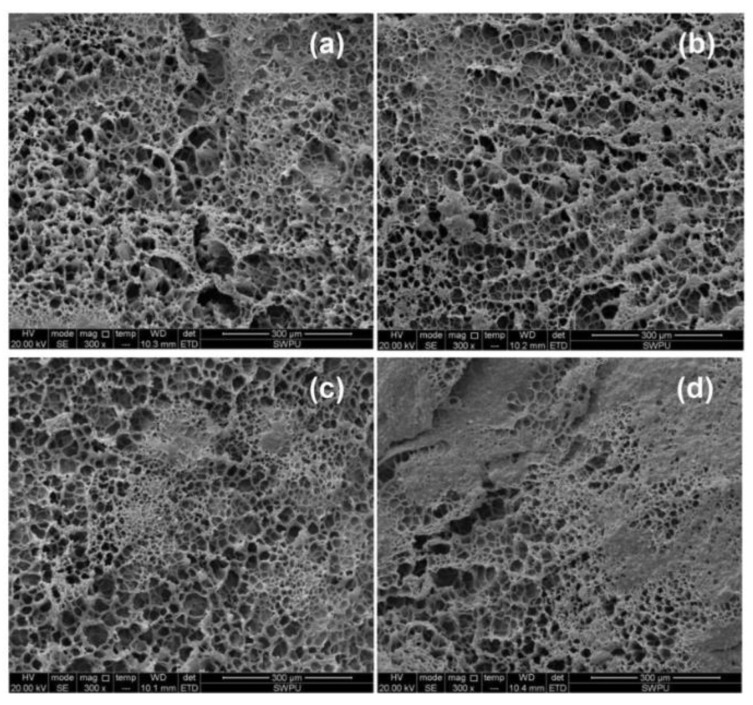
Microstructure of the polymer gel with the addition of different concentrations of SiO_2_. (**a**) 0% nano-silica; (**b**) 1% nano-silica; (**c**) 3% nano-silica; (**d**) 5% nano-silica. (Reprinted/adapted with permission from Ref. [62]. 2020, Jia, et al.).

The filling of high-temperature-resistant solid materials (e.g., clay, nanoparticles, fibers, and fruit shells) during polymer gel synthesis can also improve the high-temperature stability of the polymer gel to a certain extent. Nanoparticles have advantages of small particle size, large specific surface area, and high surface activity, and they have been widely used in oil-gas drilling and production engineering in recent years. Nanomaterials can be added as property modifiers to gel components to increase the degree of crosslinking between the polymer molecules and densify the network structure, thus optimizing the temperature resistance of the gel. Zolfaghari et al. found that nano-montmorillonite was a suitable temperature-resistant additive [60]. The hydroxyl groups (-OH) on the nanoparticle surfaces could form hydrogen bonds with the amide side chains (-CONH_2_) on the polymer molecular chains. However, the metal ions (Ca^2+^ and Al^3+^) on the clay particle surfaces could be complex with carboxyl (-COOH) oxygen on the polymer molecular chains. Both reactions enhanced the 3D network structure of the gel, improving its temperature resistance. In addition, nano-montmorillonite could also assemble together in the gel structure, forming an armor that acted as an insulator against high temperatures and inhibited the high-temperature degradation of the gel. Almoshin et al. prepared an in situ crosslinked gel system by reacting zirconia/graphene nanocomposites with low molecular weight PAM and showed that the addition of zirconia/graphene enabled the gel to form a honeycomb microscopic network structure with a smooth surface [61]. This allowed the gel to lock the internal water molecules at high temperatures. Nano-silica (SiO_2_) has also been commonly used as a solid additive for gels. SiO_2_ can be used to fill voids in a 3D network gel structure, to increase the strength of the gel skeleton. However, SiO_2_ can also bind to the hydrophilic groups on a gel skeleton through hydrogen bonding, increasing the compactness of the gel network structure (Figure 9). Jia et al. introduced nano-silica into the composite gel agent for lost circulation control, and the thermal stability of the gel increased up to 38% [62]. Patil et al. found that small-sized SiO_2_ could increase the interfacial area of the gel and subsequently increase the surface free energy, which was conducive to enhancing the temperature resistance of the gel [63]. However, filling nanomaterials for polymer gels has been mainly based on the principle of physical toughening to improve the high-temperature stability of gels. Thus, solving the problem of high-temperature polymer gel failure is still a challenge.

In general, to improve the high-temperature stability of polymer gel materials, current studies have focused on the preparation of high-temperature-resistant polymers and crosslinkers and the filling of high-temperature-resistant solid materials in the gel structure. It will be necessary to carry out in-depth research and development of new high-temperature-resistant polymers and crosslinkers from the perspective of their molecular design. Considering that solid filling materials can improve high-temperature gel stability to a certain extent, it will also be necessary to improve research on the interaction mechanisms between the gel components and the solid particles to provide a reference for the further selection of solid filling materials with excellent properties (Table 1).

## 4. Improving Salt Resistance of Polymer Gels

Oil-gas field formation water contains salt ions with concentrations ranging from a few thousand to several hundred thousand ppm. For polymer gels, high metal ion concentrations (especially high-valent metal ions) will cause the gel structure to warp and undergo syneresis by compressing the hydrophilic groups on the polymer molecular chains, thus significantly reducing gel strength.

When water salinity is high, -COOH will tend to complex with calcium and magnesium ions in the water, forming a precipitate, which will affect the gel strength, and the presence of salts will cause gel syneresis and weaken its performance. Salinity is often high in some oilfields, which will affect the application results of the gel. Therefore, research on the salt resistance of polymer gelling agents is often conducted to optimize the salt resistance of gels in terms of the polymer structure, polymer molecular weight, and the introduction of salt-resistant monomers/inorganic fillers [64].

The unsatisfactory salt resistance of conventional high molecular weight PAM (HPAM) gels is mainly because high ionic concentrations can severely compress the double diffusion layer on the carboxyl and amide group surfaces on the HPAM molecular chain, forcing the molecular chains to curl and shrink. In addition, high-valent cations (e.g., Ca^2+^ and Fe^3+^) are prone to complexing with carboxyl groups, reducing crosslinking sites, and decreasing the stability of the gel [15]. Low molecular weight PAM has better deformability and solubility than high molecular weight PAM (generally greater than 10,000 kDa) and can maintain a suitable shape of the molecular chain without excessive curling under high-salinity conditions. In addition, low molecular weight PAM has more intermolecular crosslinking with the crosslinker, increasing the crosslinking density. Fang et al. prepared a high-salt-resistant gel system using low molecular weight HPAM (molecular weight was 3800 kDa) as the polymer and HMTA/HQ as the crosslinker, and the gel had a plugging rate of over 90% for high-permeability cores even, at a high salinity of 19.8 × 104 mg/L [65].

**Figure 10:**
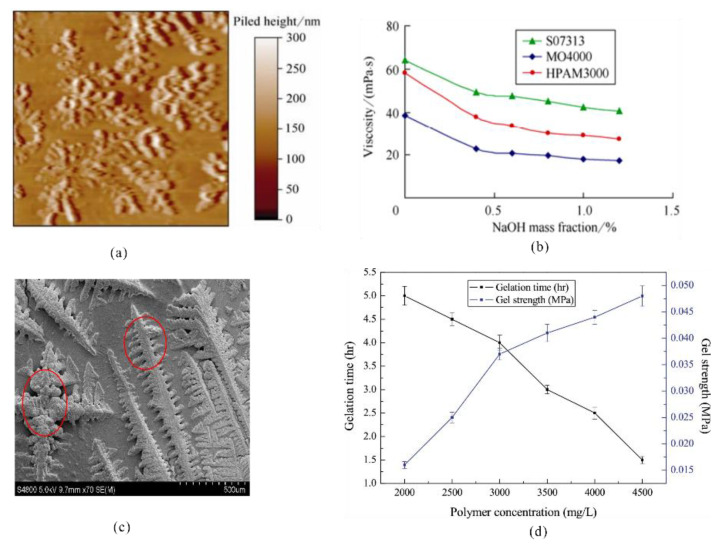
Microstructures of star/comb polymer gels and variations in their properties at different salinities. (**a**) Microscopic image of a star polymer; (**b**) effect of salinity on star polymer viscosity; (**c**) microscopic image of a comb polymer gel; (**d**) crosslinking time and strength of the comb polymer gel at different salinities (Reprinted/adapted with permission from Refs. [66,68]. 2016/2019, Luo, Yang, et al.).

The molecular structures of polymers have an important influence on gel properties. Conventional linear polymers are prone to curling under high-salinity conditions. Star polymers consist of a star-shaped core and multiple supramolecular polymer chains. This polymer has a special arm structure that enhances intermolecular chain entanglement and is prone to intramolecular associations in aqueous solutions, making it has higher salt resistance than linear polymers [66]. Cai et al. prepared a salt-resistant star polymer, Star-PAM, through copolymerization of β-cyclodextrin-grafted functional monomer with AM, followed by hydrolysis [67]. In a subsequent study, the researchers prepared a temperature and salt-resistant gel system for profile control and displacement by crosslinking the above polymer with a high-temperature-resistant crosslinker. This gel had a viscosity that reached 43,500 mPa·s after 180 days at a temperature of 126 °C and a salinity of 117,000 mg/L. In addition, composite imbibition technology combined this gel with amphoteric surfactants, and it was tested in high-temperature and high-salinity reservoirs, which showed that cumulative crude oil production increased by 3660 tons. Yang et al. found that intermolecular and intramolecular entanglements and curling were reduced through mutual repulsion between the lipophilic and hydrophilic groups on the polymer molecular chains [68]. For example, a comb-shaped arrangement of polymer molecular chains could increase the rigidity of the molecular chains and the hydrodynamic radius of the polymer chains, which significantly improved the salt resistance properties of the gel. Based on the above principle, Yang et al. prepared a gel system with a comb polymer and organic chromium crosslinker, and this system remained stable after 60 days under high-salinity (NaCl 80,000 mg/L) and high-temperature (85 °C) conditions (Figure 10) [69].

Gels prepared directly by the polymerization or crosslinking of monomers with salt-resistant functional groups typically have better salt resistance due to the denser 3D network structure and more uniform distribution of the salt-resistant functional groups in the gel backbone. Saghafi et al. prepared a PPG using four polymer monomers—namely, N,N-dimethylacrylamide (MBA), AMPS sodium salt (AMPS-Na), AM, and NVP—with MBA as the crosslinker and sodium-based montmorillonite introduced as the filler. AMPS-Na contains sulfonic acid groups with strong hydration ability, strong polarity, and can complex with multivalent cations, thus protecting the gels [69]. NVP has a five-membered ring structure and large steric hindrance, and the introduction of NVP inhibits the hydrolysis of the amide group of the polymer side chain. Furthermore, nanoparticles can significantly increase the specific surface area of the gel skeleton and enhance the salt resistance of the gel. The synergistic effect of the combined above application enabled the system to remain stable for 120 days at a high temperature of 145 °C and high salinity of 225,000 mg/L.

**Figure 11:**
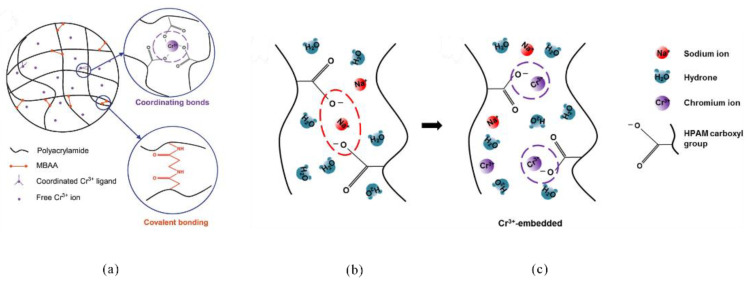
**The** Schematic diagram of the preparation of salt-insensitive microgels by pre-embedding of Cr^3+^ ligands. (**a**) The schematic representation of the Cr^3+^-embedded microgels; (**b**,**c**) schematic representations of the poly (acrylic acid)-based microgels and the Cr^3+^-embedded microgels influenced by the sodium ions. (Reprinted/adapted with permission from Ref. [70]. 2019, Pu, et al.).

Although metal ion-based crosslinked gels are prone to hydrolysis at high temperatures, resulting in gel syneresis, they are not as stable as organic crosslinked gels under high-temperature conditions. However, studies have found that the salt resistance of gels formed by the crosslinking reaction between the polymer and organic crosslinker could be significantly improved by pre-embedding the metal ions into the polymer molecules. Pu et al. synthesized a salt-insensitive microgel by free-radical suspension polymerization, in which Cr^3+^ was pre-embedded in AM and crosslinked with MBAA [70]. The embedded Cr^3+^ ligand pre-introduced the charged electrons into the gel network to inhibit the penetration of salt into the microgel, which reduced the ion shielding effect of salt on the carboxylic acid groups, thus substantially lowering the sensitivity of the gel to salt (Figure 11).

## 5. Improving Rheological and Mechanical Properties of Polymer Gels

In oil-gas drilling and production engineering, one of the main uses of polymer gels is to plug pores and fractures in formations and resolve problems such as drilling fluid loss during drilling and water channeling during production. After gel plugging of the target formation, the gel structure will be quickly destroyed under the actions of formation pressure and water scouring. Therefore, the mechanical properties of the gel will play a decisive role in their plugging effectiveness in the formation, and the rheological and mechanical properties are the two most commonly used parameters for evaluating the mechanical performance of a gel. The mechanical properties of a gel depend mainly on the polymer, crosslinker, filler material type, and concentration. They are susceptible to the influence of the formation environment, such as temperature and pressure [69,71].

**Figure 12:**
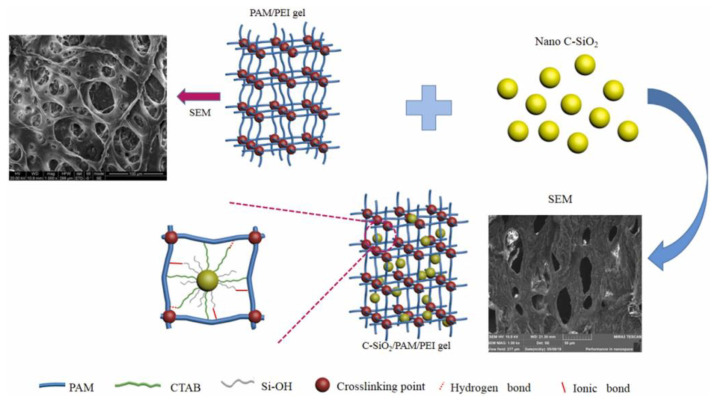
The schematic diagram of mechanism of C-SiO_2_/PAM/PEI withstands long-term stability. (Reprinted/adapted with permission from Ref. [73]. 2022, Li, et al.).

Gel component type and concentration will most intuitively influence the mechanical properties of the gel. The polymer and crosslinker type and concentration will determine the crosslinking sites that can react, thereby affecting the formation of the 3D network structure of the gel. Tessarolli et al. prepared a gel system for water shutoff based on an AMPS-NVP-PAM copolymer, PEI, and bentonite and evaluated the influence of each factor on the rheological properties of the gel [72]. Increasing the AMPS and AM concentrations also increased the storage modulus and strength of the gel, while increasing the NVP components or clay content increased the compactness of the gel grid, which increased the gel strength, and the post-gelation composite modulus reached 20–40 Pa. Li et al. prepared a polymer gel with nano-silica as the reinforcing agent (Figure 12). [73]. The study showed that enhancing gel strength is the main reason for the excellent water plugging effect and oil recovery of the C-SiO_2_/PAM/PEI gel.

Multiple crosslinked chemical bonds can also be used to construct a gel network to improve the mechanical properties of the gel. Multiple crosslinks can form an interpenetrating composite network, densifying the gel network. Jia et al. developed a multi-crosslinked gel system based on a high-temperature-resistant AM-based polymer (sulfonated polyacrylamide or SPAM), chromium acetate, and PEI [74]. Because the crosslinking rate of SPAM with chromium acetate was much higher than with PEI, SPAM was initially crosslinked with Cr^3+^ to form a weakly crosslinked gel with suitable injectability and viscoelasticity. This was conducive to injecting it into the formation, draining the formation water from the pores and fractures, and maintaining the gel component stability. After entering the target formation, the gel was crosslinked with PEI again to form a strong covalent bond network, and the presence of the double network ensured the excellent injectability and suitable gel strength of the gel system. In addition to using PAM-based polymers, modified starch has also been commonly used as a raw material for gel preparation. Gels based on modified starch are characterized by low initial and high gel viscosities, which are beneficial for achieving a suitable plugging performance [75,76]. On this basis, Zhao et al. prepared a modified starch gel system with suitable mechanical strength, suitable injectability, and an initial viscosity below 60 mPas [77]. The post-gelation modulus and viscosity of the gel increased with increasing modified starch content, and the pressure-bearing strength was as high as 24.9 MPa for fractures that were 0.42 mm wide.

**Figure 13:**
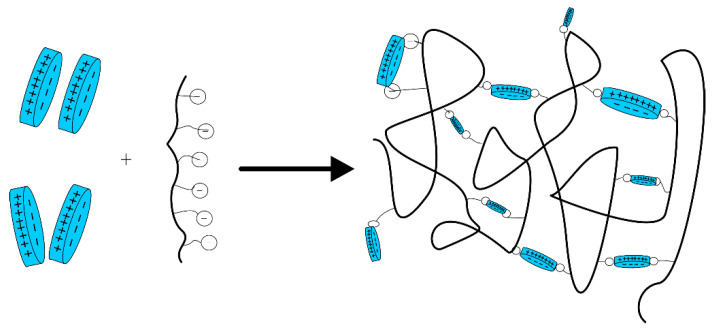
Mechanism of interaction between the anionic polymer and Li-bentonite surface cations [78]. (Adapted from Ref. [78]. 2012, Tongwa, et al.).

Adding inorganic materials such as nano-silica and clay (e.g., bentonite and Li-bentonite) to the gel system can considerably improve the mechanical properties of the gel through physical filling, chemical reaction, or the synergistic effect of nano-silica and clay. Tongwa et al. prepared a Li-bentonite-enhanced nanocomposite hydrogel based on anionic partially hydrolyzed AM [78]. The main principle was as follows. First, the negatively charged polymer molecular chains would bind to the edges of the positively charged Li-bentonite sheets, and second, the hydroxyl groups (-OH) on the surface of Li-bentonite would interact with the amide groups (-CONH_2_) on the amide side chains of the polymer, forming hydrogen bonds. In addition, the cations on the Li-bentonite surface would form complexes with the carboxyl groups of the polymer (Figure 13). The experimental results showed that the elastic modulus of the gel increased by approximately 30,000 Pa as the Li-bentonite concentration in the gel components increased. Mohamadian et al. prepared a hybrid nanocomposite of poly(styrene-methyl methacrylate acrylic acid)/clay as a novel rheology-improvement additive for drilling fluids. The new drilling fluids maintained fluid-loss control properties to temperatures up to about 200 °F and salinities of up to about 40 g/L NaCl concentration and reduced fluid loss by about 77% for the terpolymer-treated drilling fluid [79,80].

**Figure 14:**
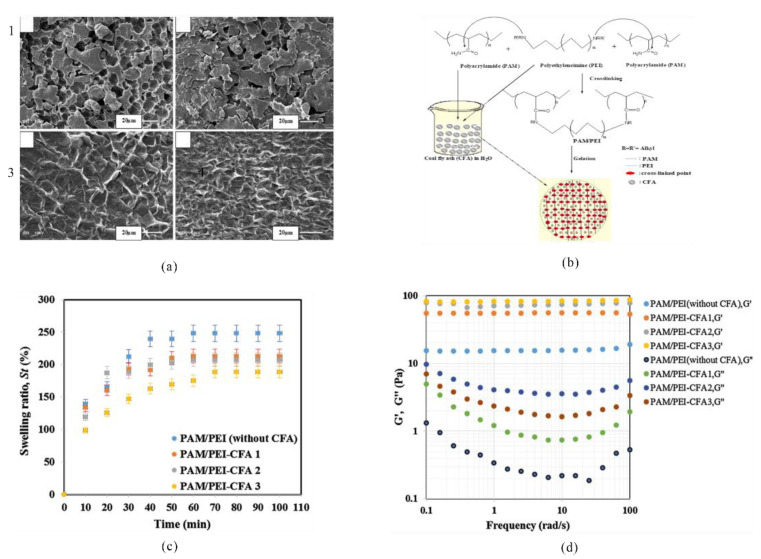
Comparison of preparation principle and performance of PAM/PEI and PAM/PEI-CFA gels. (**a**) SEM images of hydrogels with different CFA content; (**b**) synthesis route of the AM/PEI-CFA hydrogel; (**c**,**d**) swelling and rheological properties of hydrogels with different CFA contents. (Reprinted/adapted with permission from Ref. [81]. 2018, Adewunmi, et al.).

Adewunmi et al. prepared PAM/PEI and PAM/PEI-CFA gels using PEI as the crosslinker and coal fly ash (CFA) as the inorganic filler as filling materials [81]. The CFA was mainly composed of alumina and calcium oxide. The experimental results showed that the gel with CFA addition exhibited a larger elastic modulus and a denser network structure when observed under an SEM (Figure 14).

**Figure 15:**
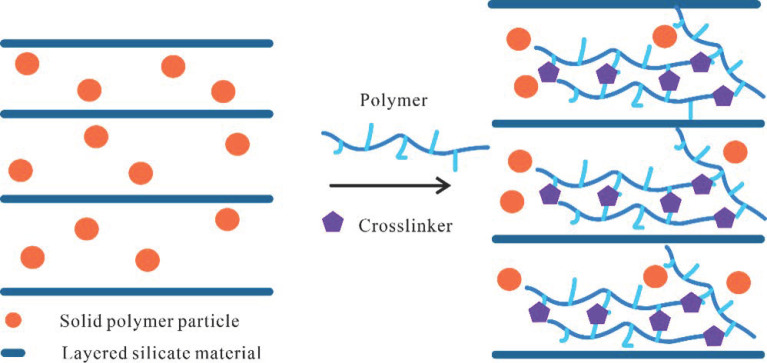
Preparation principle of intercalation polymerization of a composite gel and physical photographs of the compressive test. (Adapted from Ref. [82]. 2020, Jia, et al.).

Conventional inorganic nanomaterials will improve the mechanical properties of gels mainly through physical embedment. The gel properties can be significantly improved by combining the active nanomaterials with the gel structure through chemical methods, and intercalation polymerization is a commonly used method. First, the modified layered silicate inorganic minerals are dispersed in the monomers by intercalation polymerization, allowing some monomers to enter the interlayers. Then, the monomers will start to polymerize and crosslink, thereby forming a gel with suitable mechanical properties. Using the above principle, Jia et al. [82] developed a composite gel material with a storage modulus of 15 kPa and compressive strength of 8.5 MPa at 160 °C (Figure 15). The above intercalation polymerization method could serve as an inspiration for the development of highly elastic, high-strength gels.

## 6. Conclusions

Because of suitable viscoelasticity and deformability, polymer gels have been widely used for formation plugging, lost circulation control, profile control, and water shutoff in oil-gas engineering. Commonly used polymer gels can be categorized as in situ crosslinked gel and ground pre-crosslinked gel. According to the different formation conditions, the two kinds of polymer gels have different synthesis principles and preparation methods, and their temperature resistance, salt resistance, and mechanical properties are also different. The main advantage of in situ crosslinked polymer gel is that it can generate a high-strength bulk gel in in situ pores and fractures to achieve a high-strength plugging zone, and the main advantage of ground pre-crosslinked gel is that its particle size can be selected according to the size of pores and fractures, and it is less affected by formation temperature, salinity, and shear action.

To expand the application range of polymer gels in response to high temperature and high salinity formation, we reviewed the strategies for increasing the high-temperature resistance, high salt resistance, gelation rheological, and mechanical strengths of polymer gels in terms of improving the polymer, crosslinker, and additive structures and types. Meanwhile, the action mechanisms of polymer gels for profile control and water shutoff in oil-gas reservoirs, in-depth profile control and displacement in formations, and lost circulation control during drilling were also analyzed. It provides theoretical and technical references for the development and optimization of polymer gels that may be suitable for different oil-gas drilling and production processes.

## Figures and Tables

**Table 1:** Methods to improve the temperature resistance of polymer gel.

Strategy	Principle	Measure	Molecular Structure
Optimize polymer molecular structure	Introduce functional monomers (large side groups/rigid side groups, and hydrolysis-resistant/inhibiting amide monomers)	Introduce functional monomers and terpolymers (e.g., NVP and AMPS)	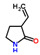 (NVP) 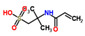 (AMPS) 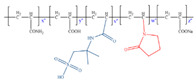 (AM-AMPS-NVP) copolymer
Select the appropriate type of chemical crosslinker	Use crosslinker with a cyclic structure to form temperature-resistant chemical bonds	Phenol/formaldehyde crosslinking, HQ/HMTA crosslinking system, PEI (PEI)	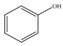 (Phenol) 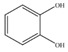 (catechol) 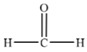 (Formaldehyde) 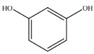 (Resorcinol) 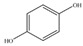 (HQ) 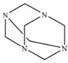 (HMTA) 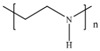 (PEI)
Optimize the gel network structure	Form an interpenetrating network structure in the gel to densify the 3D network of the gel	Introduce nanomaterials (e.g., nano-montmorillonite, nano-silica), use composite crosslinkers	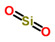 (Nano-silica)

## Data Availability

All persons included have agreed to confirm.
